# RNA sequencing of blood from sex- and age-matched discordant siblings supports immune and transcriptional dysregulation in autism spectrum disorder

**DOI:** 10.1038/s41598-023-27378-w

**Published:** 2023-01-16

**Authors:** Pasquale Tomaiuolo, Ignazio Stefano Piras, Simona Baghai Sain, Chiara Picinelli, Marco Baccarin, Paola Castronovo, Marco J. Morelli, Dejan Lazarevic, Maria Luisa Scattoni, Giovanni Tonon, Antonio M. Persico

**Affiliations:** 1 Mafalda Luce Center for Pervasive Developmental Disorders, Milan, Italy; 2 Neurogenomics Division, The Translational Genomics Research Institute, Phoenix, AZ USA; 3 Center for Translational Genomics and Bioinformatics, IRCCS San Raffaele Scientific Institute, Milan, Italy; 4 Department of Genetics, Synlab Suisse SA, Bioggio, Switzerland; 5 Research Coordination and Support Service, Istituto Superiore di Sanità, Rome, Italy; 6 Child and Adolescent Neuropsychiatry Program, Department of Biomedical, Metabolic and Neural Sciences, University of Modena and Reggio Emilia, Via Giuseppe Campi 287, 41125 Modena, Italy

**Keywords:** Genetics, Biomarkers, Molecular medicine

## Abstract

Autism spectrum disorder (ASD) is a neurodevelopmental condition with onset in early childhood, still diagnosed only through clinical observation due to the lack of laboratory biomarkers. Early detection strategies would be especially useful in screening high-risk newborn siblings of children already diagnosed with ASD. We performed RNA sequencing on peripheral blood, comparing 27 pairs of ASD children vs their sex- and age-matched unaffected siblings. Differential gene expression profiling, performed applying an unpaired model found two immune genes, *EGR1* and *IGKV3D-15*, significantly upregulated in ASD patients (both *p* adj = 0.037). Weighted gene correlation network analysis identified 18 co-expressed modules. One of these modules was downregulated among autistic individuals (*p* = 0.035) and a ROC curve using its eigengene values yielded an AUC of 0.62*.* Genes in this module are primarily involved in transcriptional control and its hub gene, *RACK1*, encodes for a signaling protein critical for neurodevelopment and innate immunity, whose expression is influenced by various hormones and known "endocrine disruptors". These results indicate that transcriptomic biomarkers can contribute to the sensitivity of an intra-familial multimarker panel for ASD and provide further evidence that neurodevelopment, innate immunity and transcriptional regulation are key to ASD pathogenesis.

## Introduction

Autism spectrum disorder (ASD) is a multifactorial neurodevelopmental disorder with onset in early childhood, characterized by social and communication deficits, stereotyped behaviors, restricted interests, and abnormal sensory processing^1^. ASD affects about 1% of the population, ranging from 1/54 children and 1/45 adults in the United States^2,3^, to 1/87 children in Italy and 1/102 adults in England^4,5^. ASD is considered one of the behavioral disorders with the highest genetic component^6,7^. Heritability, or the amount of variability of a certain character attributable to genetic factors, varies from 50 to 95% in ASD^8^. Furthermore, estimates of disease risk among siblings of ASD patients range from 3 to 18%, values well above the prevalence observed in the general population^9,10^. Submicroscopic cytogenetic abnormalities called copy number variations (CNVs) can contribute to autism in approximately 5–10% of the cases^7,11^. Despite the lack of a clear or universal genetic mechanism, several studies have revealed specific genetic factors for isolated cases as well as a number of candidate genes and chromosomal regions indicated as relevant across multiple studies, such as 1q21.1, 2q13, 2p16, 3q29, 7q11.23, 15q11.2-q13.1, 16p11.2, 22q13.3^12,13^.

Transcriptomic studies are a key link between protein levels and genetic factors. RNA expression studies are a useful tool to characterize complex human diseases and predict associated molecular and cellular processes^14^. Early transcriptome studies in ASD were conducted using post-mortem brain tissue from ASD patients^15–18^. RNA obtained from peripheral blood mononuclear cells (PBMCs) subsequently became of interest in autism research, because blood collection is characterized by low invasiveness, ease of use, and cost-effectiveness. ASD is still diagnosed exclusively based on clinical observation and investigators are actively seeking laboratory biomarkers able to aid clinicians in early diagnoses, prognostic predictions, and targeted choice of therapeutic strategies^19^. Blood-based biomarkers could be used for large-scale screening and to support diagnosis in clinical settings^19^. Several studies were thus performed on blood allowing the identification of genes and pathways dysregulated in autistic individuals. Initially, Hu et al.^20^ analyzed RNA extracted from lymphoblastoid cell lines (LCLs) obtained from 3 pairs of monozygotic twins discordant for ASD. They identified 25 up-regulated and 19 downregulated genes including 8 genes associated with neurological functions, development, or diseases. The same group conducted a second study^21^ performing microarray analysis on 116 LCLs from ASD patients stratified into three phenotypic categories and compared to age-matched controls. They identified 530 genes differentially expressed between ASD patients and controls. These genes were enriched for synaptic transmission, neurogenesis, neurulation, long-term potentiation, protein ubiquitination, and brain function. Additionally, only in the most severely affected ASD subgroup, 15 differentially expressed genes were associated with the regulation of the circadian rhythm^21^. Other studies provided evidence of the involvement of the immune system in a large subset of patients with ASD. In one of the first transcriptomics studies conducted on blood, Gregg et al.^22^ selected patients with a history of developmental regression or history of early-onset and controls from the general population. No genes were significantly dysregulated between ASD and controls. However, 11 genes expressed in natural killer cells (NK) and enriched for the NK cytotoxicity pathway were shared among all ASD patients compared to controls. Enstrom et al.^23^ then analyzed peripheral blood from 35 children with ASD and 11 age- and gender-matched controls confirming an enrichment of the expression of genes correlated with NK cells function. This suggests that abnormalities in NK cells may represent a susceptibility factor for ASD, predisposing to the development of autoimmunity or to adverse neuroimmune interactions during a critical period of neurodevelopment. Interestingly, a possible decreased NK cell function in ASD was already reported in 1987 by Warren et al.^24^ Meanwhile, weighted gene correlation network analysis (WGCNA) analysis^25^ has become a widely used approach both in brain- and blood-based studies of ASD, allowing the identification of modules encompassing functionally-related, co-expressed genes^17,26–28^. Using this method, relevant roles for NK and naïve B cells were recently confirmed by Filosi et al.^26^, comparing 75 discordant sibling pairs. Modest, yet significant transcriptomic differences were detected mainly reflecting changes in peripheral blood immune genes, supporting possible roles for *NMUR1*, *HMGB3*, and *PTPRN2*, at the single gene level, and of “NK cell mediated cytotoxicity”, and “Immunoregulatory interactions between a lymphoid and a non-lymphoid cell” as different modules between affected and unaffected siblings^26^. Collectively, much data supports the idea that transcriptomic profiling of peripheral blood may provide candidate biomarkers for ASD and that immune dysregulation may play a relevant role in ASD pathogenesis. However, a consistent set of diagnostic biomarkers remains elusive, likely because of ASD heterogeneity and to methodological issues. For example, some studies lack sex matching between ASD discordant siblings^26,29^, and sex has been shown to significantly influence peripheral transcriptomics in ASD^30^.

The present study uses an intra-familial design, matching affected and unaffected sibling pairs by sex and age, while controlling for pharmacological treatment in the autistic sibling, an important confounding factor often overlooked in prior Literature. We thus conducted RNA sequencing (RNA-Seq) in 31 ASD and 31 unaffected siblings (SIB) matched by sex and age, later reduced to 27 ASD-SIB pairs to reliably control for pharmacological treatment. Its aim is to find ASD blood biomarkers possibly applicable within “high risk” families, where one child has already received an ASD diagnosis.

## Results

### Identification and control of confounding variables

We first conducted two exploratory analyses, to identify potential confounding factors: (a) a Principal Component Analysis (PCA) analysis detected a batch effect, due to the processing of the samples in two different runs, in the absence of outliers (Supplementary Fig. S1A); and (b) a differential gene expression (DGE) analysis detected a sizable effect exerted by psychoactive drugs, taken by 10/27 (37.0%) ASD cases at the time of blood collection, resulting in 17 genes significantly upregulated and 20 genes significantly downregulated (Supplementary Fig. S1B and Supplementary_gene_list_drug_effects_ASD file). Four ASD-SIB pairs were subsequently excluded, because compliance with prescribed drug treatment was uncertain. All further analyses were carried out on 27 ASD-SIB pairs, controlling for batch and for presence/absence of pharmacological treatment. Demographic and clinical characteristics of these 27 ASD-SIB pairs are summarized in Table 1.

**Table 1:** Demographic and clinical characteristics of the 27 pairs of autistic and unaffected siblings recruited in this study.

Characteristics	N (%)
**Sex**	
Male	23 (85.2%)
Female	4 (14.8%)
M:F	5.75:1
**Mean age ± SD in years (range)**	
ASD	11.32 ± 8.38 (2–32)
Unaffected siblings	11.88 ± 8.04 (2–29)
**Family type**	
Simplex	25 (92.6%)
Multiplex	2 (7.4%)
**Pharmacological treatment**	
Present	10 (37.1%)
Absent	17 (62.9%)
**Expressive language**	
Normal development	6 (22.3%)
Language delay	7 (25.9%)
Loss after normal development	7 (25.9%)
Never acquired	7 (25.9%)
**Intellectual disability**	
Present	18 (66.7%)
Absent	9 (33.3%)

### Differential gene expression analysis

**Figure 1:**
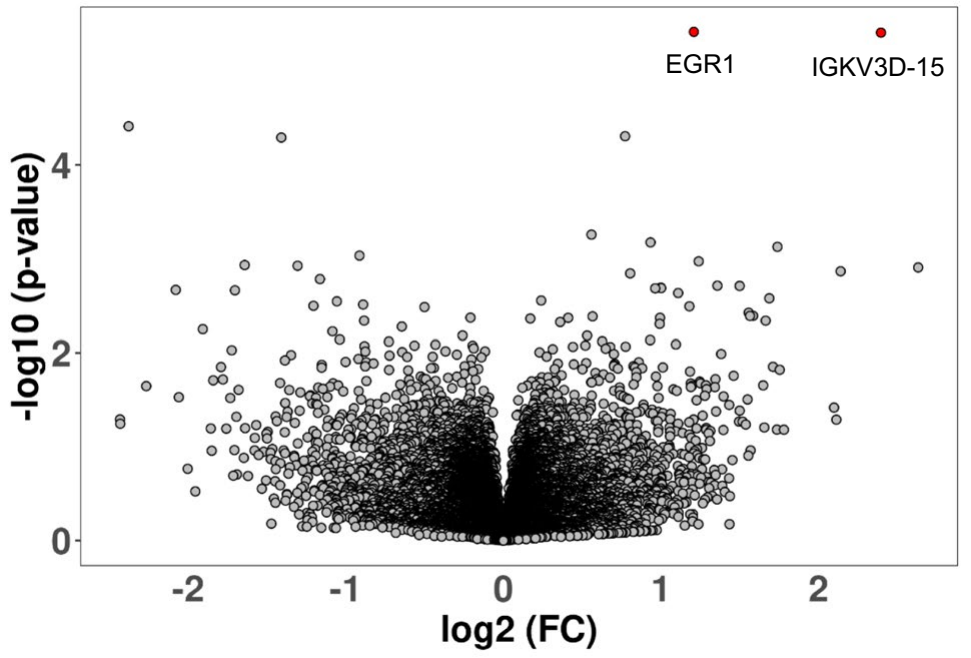
Volcano plot of differentially expressed genes using the unpaired model. Significantly overexpressed genes, *EGR1* and *IGKV3D-15*, are highlighted in red.

**Figure 2:**
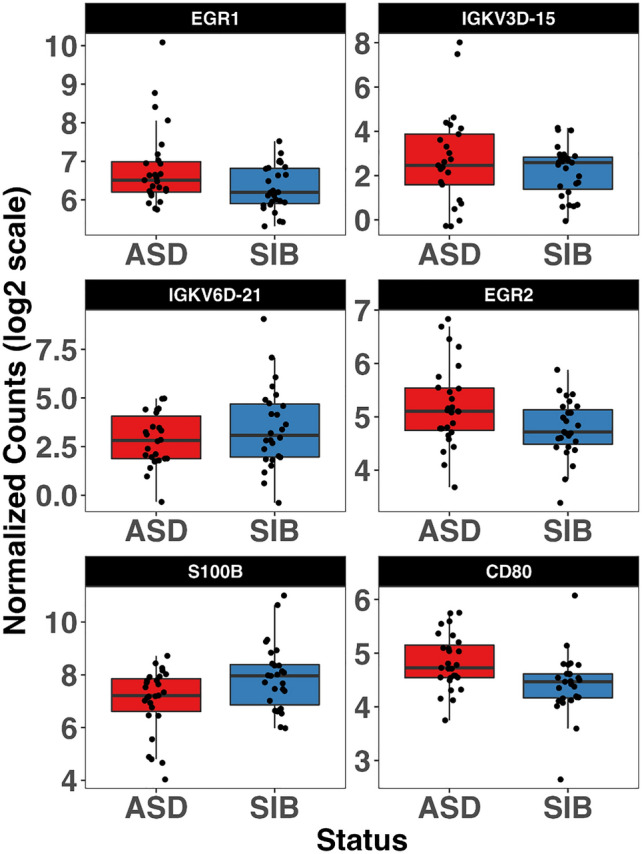
Boxplot of the top six differentially-expressed genes detected using the unpaired model. *EGR1* and *IGKV3D-15* retain significant *p* values after FDR correction (both *p* adj = 0.037).

To test for ASD-SIB expression differences, we performed DGE analysis using two distinct models: unpaired and paired. Applying the unpaired model to our sample, while correcting for sequencing run and pharmacological treatment, two genes retain a significant *p* value after FDR adjustment, namely *EGR1* (*p* adj = 0.037), and *IGKV3D-15* (*p* adj = 0.037), both up-regulated in ASD patients compared to their typically-developing siblings (Fig. 1). Furthermore, several other genes showed a nominal *p* value < 0.05, but did not reach statistical significance after FDR correction. In particular, *IGKV6D-21* (*p* value = 3.87E−5, *p* adj = 0.193) and *S100B* (*p* value = 5.10E−5, *p* adj = 0.193) were down-regulated in ASD < SIB; instead *EGR2* (*p* value = 4.94E−5, *p* adj = 0.193), and *CD80* (*p* value = 5.53E−4, *p* adj = 0.999) were up-regulated in ASD > SIB (Fig. 2). Applying the paired model, six genes achieved nominal significance (Supplementary Table S1), but none retained statistical significance after FDR correction (Supplementary Figs. S2 and S3). Log2 Fold Changes for the paired and unpaired model were significantly correlated (Supplementary Fig. S4).

### WGCNA analysis

**Figure 3:**
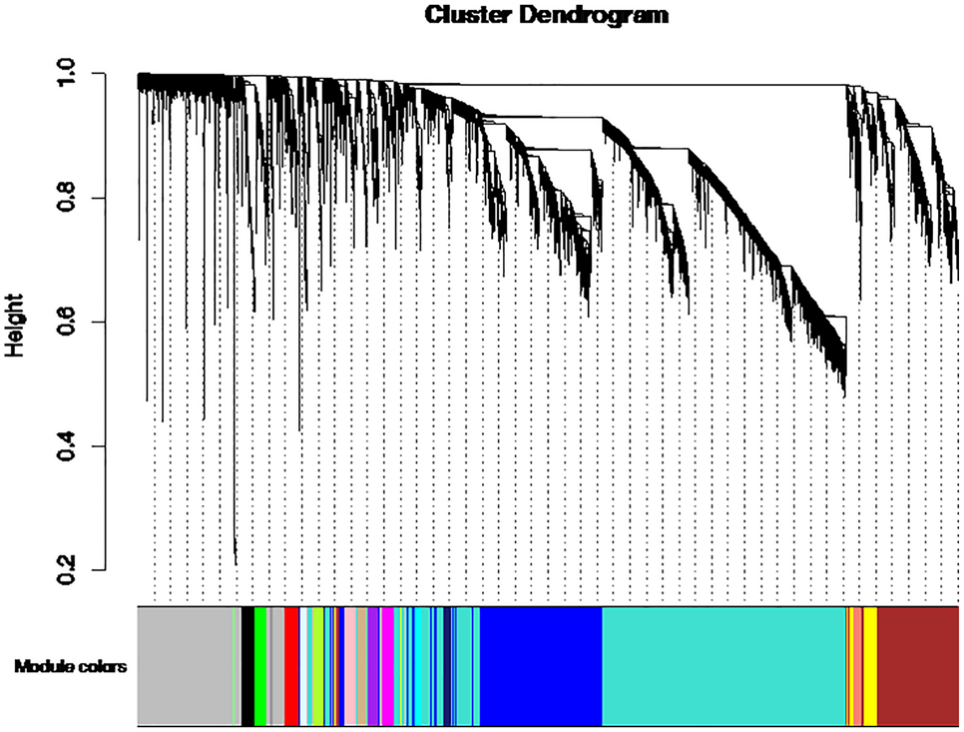
Co-expression modules. Cluster dendrogram showing the coexpression modules detected by WGCNA analysis.

After excluding low-expression and low-variability genes, WGCNA analysis was conducted on 9491 informative genes. We set the soft threshold power = 8 to construct a scale-free network. Next, we built the adjacency matrix and constructed the topological overlap matrix (see Supplementary Methods for additional information). Using this strategy, 18 modules encompassing 8095 informative genes were identified, based on average hierarchical clustering and dynamic tree clipping (Fig. 3), whereas only 1396 genes were not included in any network (grey module in Fig. 3).

**Figure 4:**
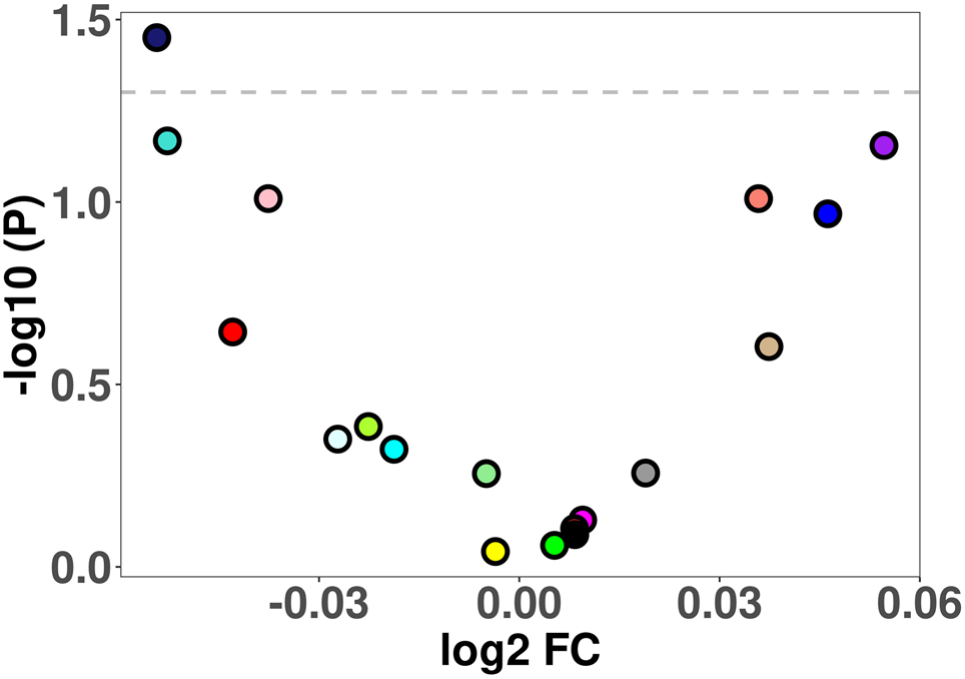
Volcano plot of differential expression of module eigengenes. Module eigengenes were obtained for each co-expression module and used for differential expression between ASD and SIB. The “midnight blue” co-expression module reached nominal significance and was downregulated in ASD patients compared to SIBs. The dashed line indicates *p* < 0.05 significance level.

Module eigengenes (first principal component of the genes included in the modules) were obtained and used for differential expression between ASD and SIB. Data were again analyzed using both the unpaired and paired models, which were highly correlated (Supplementary Fig. S4). Applying the unpaired model, one co-expression module reached nominal significance before multiple test correction (midnight blue; *p* = 0.035; log2FC = − 0.05), and was downregulated in ASD patients (Fig. 4). In addition to the “midnight blue” module, suggestive trends (unadjusted *p* < 0.10) are displayed by the “turquoise” (primary metabolic process) and “pink” (wound healing) modules towards downregulation, and by the “purple” (B cell receptor signaling pathway) and “salmon” (innate immune response) modules toward upregulation (Fig. 4 and Supplementary Fig. S5).

**Figure 5:**
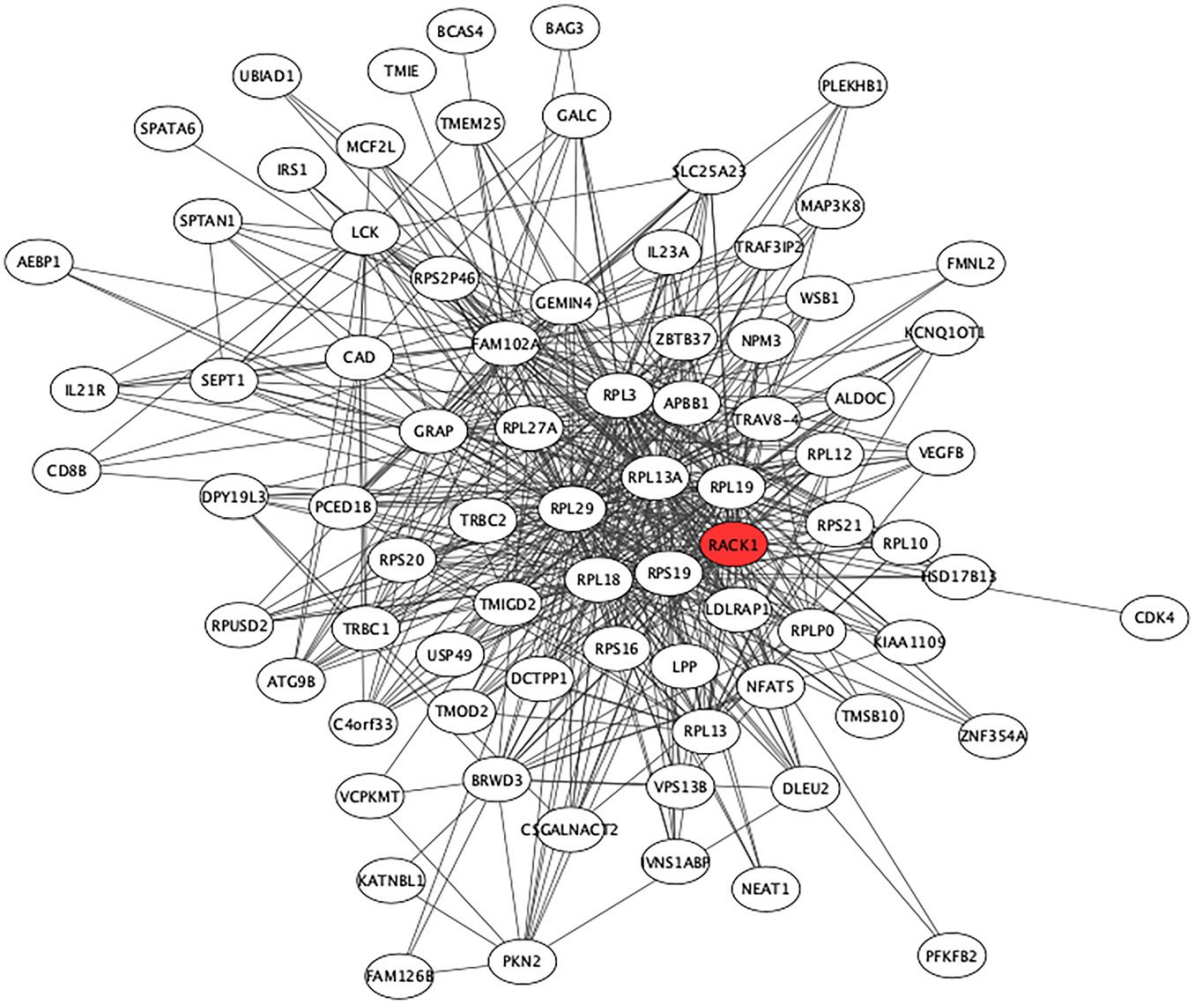
The “midnight blue” coexpression network. The figure was generated using using Cytoscape^31^, and the hub gene *RACK*1 is highlighted in red.

**Figure 6:**
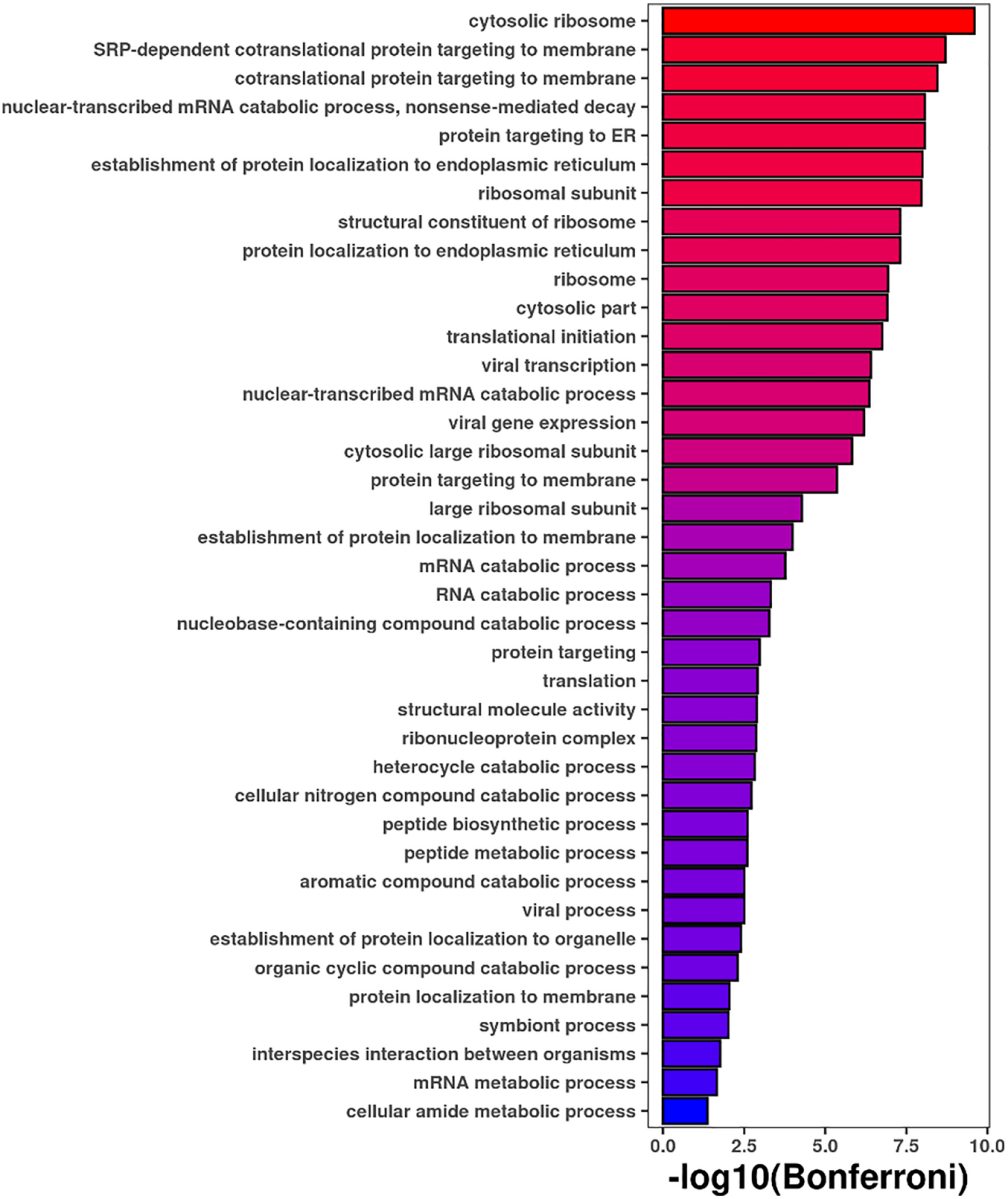
Gene ontology analysis of the “Midnight blue” module (n = 82 genes). Only processes significant after Bonferroni correction are listed, with increasing significance going from blue to red.

We visualized the co-expression network of the midnight blue module using Cytoscape^31^, exporting only edges with a weight larger than 0.10 (corresponding to the strength of correlation between modules). The hub gene of the network is *RACK1,* a scaffolding protein involved in the recruitment, assembly and/or regulation of a variety of molecules, belonging to the PKC, Src and MAPK signaling pathways (Fig. 5). Gene Ontology enrichment analysis using all 82 genes included in the midnight blue module detected an enrichment in transcription- and translation-related biological processes, with ASD > SIB in cytosolic ribosome, SRP-dependent cotranslational protein targeting to membrane and protein targeting to ER, whereas ASD < SIB in various catabolic processes, such as nuclear-transcribed mRNA catabolic process (Fig. 6). To test the specificity and the sensitivity of this model, we computed a ROC curve using the values of eigengene (midnight blue), obtaining AUC = 0.62 (Supplementary Fig. S6).

## Discussion

Autism is a complex disorder, whose phenotypic expression in most cases seemingly depends on the combined action of genetic and environmental factors. The identification of its pathogenetic bases is complicated by the presence of great genetic and phenotypic heterogeneity. Transcriptomics represents an intermediate level between gene and function, potentially able to provide useful information in the search for ASD biomarkers. In this work, we conducted a transcriptome-wide study using RNA-seq technology on an ethnically homogeneous sample consisting of 27 Italian autistic individuals paired with their unaffected sibling, matched by sex and age. Moreover, psychopharmacological treatment at the time of blood drawing was carefully considered and statistically controlled.

Differential expression analysis identified some significant differences between ASD patients and their unaffected siblings. Indeed, two genes were significantly upregulated in ASD, *EGR1* and *IGKV3D-15*. Interestingly, both genes play important immune roles in peripheral mononuclear blood cells (PMBCs). *EGR1* (early growth response protein 1), also known as Zif-268^32,33^, encodes for a member of the zinc-finger family of transcription factors. Its expression is stimulated by a variety of stimuli (growth factors, hormones, endotoxins, microbial infections, UV, hypoxia, mechanical stress and/or injury, proinflammatory cytokines); either directly or heterodimerizing with other factors, like NGFI-A binding protein (NAB), CREB-binding protein (CBP), yes-associated protein 1 (YAP-1), p53 and NFκB. EGR-1 modulates the expression of many genes ultimately exerting proinflammatory, proliferative and migration-promoting actions in multiple systems and organs, including cardiovascular, renal, skin, gut and lung^34^. This result may have important clinical implications, considering that (a) respiratory and cardiovascular diseases are among the leading cause of premature, often sudden death especially in low-functioning autistic individuals^35^. Their pathogenesis consistently involves an inflammatory and proliferative component, coordinated by EGR-1^34^; (b) skin inflammatory disorders, like atopic dermatitis, are present in approximately 10% of children with ASD and are significantly associated with neurodevelopmental disorders^36^; and (3) EGR-1 is also strongly expressed in the gut epithelium, where it plays a complex role in regulating inflammation and immune interactions between host and microbiote^37^.

*IGKV3D-15* (Immunoglobulin kappa variable 3D-15) encodes for membrane-bound or secreted glycoproteins produced by B lymphocytes. In the recognition phase of humoral immunity, the membrane-bound immunoglobulins serve as receptors which, upon binding of a specific antigen, trigger the clonal expansion and differentiation of B lymphocytes into immunoglobulins-secreting plasma cells. Secreted immunoglobulins mediate the effector phase of humoral immunity, which results in the elimination of bound antigens^38,39^.

Biological processes like “inflammation”, “immune system regulation”, “acute phase response”, and “lipid metabolism” have already been reported to be dysregulated in prior unbiased transcriptional and proteomic studies using whole blood and serum, respectively, from ASD patients and typically developing controls^40,41^. Interestingly, genome-wide RNA expression studies generally find these genes upregulated in post-mortem brains and down-regulated in blood^40^, whereas proteomic studies instead find predominantly increased serum levels of proteins encoded by these gene networks^41^. In addition, many ASD-relevant genes are exclusively expressed in the brain and often times genes and modules display opposite expression trends in brain and whole blood^40^. These discrepancies spur several considerations. First, especially for single-gene analyses, our careful ASD-SIB matching strategy may have been instrumental, because stratification by gender, age, and ethnic group has been shown to largely influence both transcriptomic and proteomic analyses in ASD^41,42^. Secondly, our results should be primarily interpreted as reflecting pathophysiological processes occurring peripherally at the systemic level, because their relationship to the brain pathophysiology of ASD may be limited. Thirdly, despite the latter caveat, analyzing EGR-1 expression with the database dbMDEGA^43^, on the combined dataset available from Voineagu et al.^17^, Chow et al.^18^ and Ginsberg et al.^44^, collectively yields a nominal *p* value of 0.03 (FDR *p* = 0.15, n.s.), documenting a modest yet consistent increase in EGR-1 expression in the male neocortex (35 ASD vs. 49 CON), whereas the female neocortex displays the opposite trend (15 ASD vs. 18 CON). Since all neurodevelopmental disorders including ASD are male-predominant and our sample has a M:F ratio of 5.75:1 (Table 1), our findings may also be somewhat relevant for the CNS. Interestingly, EGR1 is involved in the regulation of synaptic plasticity, learning, and memory, and is considered a candidate gene for schizophrenia, bipolar disorder and major depressive disorder^45^. This immediate-early transcription factor has a distinct pattern of brain gene expression and mediates the expression of a number of late-response genes involved in different neuronal processes, from growth control to plastic changes. EGR-1 expression has been found to be upregulated by neuronal activity and synaptic stimulation in many contexts, ranging from visual plasticity in the visual cortex to the “social brain” network in social insects^46^. It thus appears as a very interesting candidate, able to bridge multiple apparently unrelated peripheral and central functions displaying abnormalities in ASD.

A novel finding in the present study is represented by the detection, using WGCNA analysis, of one module, “midnight blue”, showing a nominally significant decrease in ASD expression compared to SIB. This module appears mainly involved in protein translation (Fig. 6). Its down-regulation, albeit modest, may be functionally relevant in relation to the immune dysregulation documented by our single-gene analyses and by other studies, as well as possible negative influences on neurodevelopment. Interestingly, the hub gene of the network is *RACK1*, encoding the receptor for activated C kinase 1 (Rack1), a propeller-shaped scaffolding protein with seven WD40-repeat (WDR) domains each able to bind, assemble and regulate a variety of signaling molecules^47^. Its first binding partner ever identified was activated protein kinase CβII (PKCβII)^48^, the protein product of *PRKCB*, a strong candidate autism gene according to the SFARI GENE database (https://gene.sfari.org/). The PKCβII isoform plays an important role in driving inflammatory processes and we previously found it downregulated in ASD brains, although ASD-associated alleles do not allow this down-regulation, conceivably increasing ASD risk by further promoting neuroimmune dysregulation^49^. On the one side, the downregulation of *RACK1* and of the “midnight blue” module could thus represent a negative-feedback attempt to contrast a systemic inflammatory and immune activation^50^. At the opposite end, *RACK1* expression is directly influenced by various hormones as well as by exposure to pollutants capable of acting as "endocrine disruptors (phthalates, bisphenols, polychlorinated biphenyls, etc.)^51^. Prenatal exposure to some of these chemicals, such as phthalates, has been shown to significantly enhance ASD risk^52^. Indeed during neural development, *RACK1* regulates axon growth and guidance^53–55^, linking its altered expression or signaling to various neurodevelopmental disorders, such as fetal Down syndrome^56^, but also late-onset CNS disorders like Alzheimer’s disease display similarly altered RACK1 function^57^. Hence, if confirmed in future studies, decreased “midnight blue” expression could represent either a negative-feedback response to persistently ongoing inflammation, oxidative stress and immune dysreactivity, or a primary expression dysregulation due to prenatal exposure to endocrine disruptors during critical periods in transcriptional control determination.

This study has several strengths and limitations, which must be duly acknowledged. Controlling for ethnicity, age, and sex represents an important strength of our study design, because these variables have indeed been shown to influence the results of transcriptomic and proteomic studies using whole blood, leukocytes, or serum as starting biomaterial^41,42^. To our knowledge, careful pairwise intrafamilial matching to minimize the influence of these confounding variables has been applied by some, but not all studies applying a similar sib-pair design^26,29^. In addition, our ASD cases do not come from a repository, but were clinically followed by one of the Authors (AMP), so we could reliably assess for ongoing psychopharmacological treatment at the time of blood drawing. Controlling statistically for this parameter is extremely important, since we show here that it exerts a sizable influence on blood gene expression potentially sufficient to obscure or distort ASD-SIB differences (Supplementary Fig. S1B). Through this careful strategy we have lowered the probability of false-positive findings, which plague the ASD biomarker field producing a lack of replication and heterogeneity of results. Furthermore, an intrafamilial design is also more clinically useful in terms of biomarker search. In fact, unaffected siblings are different from unrelated typically developing controls. ASD risk in newborn siblings of children already diagnosed with ASD is indeed much higher compared to the general population, because siblings share 50% of their genome and potentially harmful environmental exposures. Moreover, first-degree relatives often display subthreshold autistic traits, leading to the term “autism spectrum” to designate this continuum of autistic traits in the general population^58^. Finally, comparing autistic children against their unaffected siblings reduces the probability of an environmental bias, due to exposure to agents differentially distributed in residential areas and households.

This increased reliability was obtained at the expense of decreased sensitivity, which represents the main limitation of this study, due to its relatively low statistical power given the available sample size. Also the 50% genetic overlap in ASD-SIB pairs, as compared to unrelated ASD-control pairs, could contribute to lower the positive predictive value, although this explanation alone may not be entirely satisfactory, as siblings should also carry differentially expressed resilience factors that could emerge from a larger sample size. Power analysis indicates that assuming the total number of genes tested is 10,000, the top 100 genes are prognostic, and the desired minimum fold change is 2, in order to reject the null hypothesis with a probability (power) of 0.7 using the exact test (FDR corrected *p* value = 0.01) a sample size of 56 ASD-SIB pairs is necessary. Indeed, our original sample included only 31 ASD-SIB pairs and it was further reduced down to 27, to enhance reliability relative to pharmacological treatment. Given these premises, it is not surprising that, after controlling for multiple testing, statistical significance was achieved only in the unpaired design, which is more powerful but also more prone to bias. Consequently, until we recruit additional pairs to reach this sample size, the present results should be viewed with caution in reference to the potential for false positives especially in single gene analyses, to the likely presence of several false-negatives and to the relatively small effect size of “midnight blue” decreased expression. Finally, our RNA sequencing was not deep enough to allow a reliable ASD-SIB differential alternative splicing analysis. Unfortunately, this is a major limitation, because abnormal alternative splicing and RNA management^27,59^, possibly linked to an altered gut microbiome^60^, represents a rapidly emerging area of interest in ASD research and may explain ASD-SIB discordance, at least in some cases.

Despite these limitations, the present study confirms and extends prior genome-wide transcriptomic findings, pointing again toward peripheral immune and transcriptional dysregulation in ASD, while providing some novel candidate single-gene and module biomarkers for future targeted studies.

## Methods

### Human samples description

We initially selected 31 pairs of siblings, each pair including one sibling with idiopathic autism (ASD) and one unaffected sibling (SIB). Participants were ethnically homogeneous (all Italians) and were enrolled at Campus Bio-Medico University Hospital, Rome, Italy and at the Interdepartmental Program “Autism 0-90”, “Gaetano Martino” University Hospital, Messina, Italy. The patient/sibling pairs were selected excluding any known comorbidities or genetic syndromes and were matched by sex and age. The use of drugs was not an exclusion criterion, but drug treatment was used as a covariate (dichotomous variable: yes/no) throughout the study, because preliminary analyses showed its significant effects on transcription patterns (Supplementary Fig. S1B). Four pairs, out of the original sample of 31 pairs, were excluded, because compliance with drug treatment was uncertain. Among the remaining 27 patients with ASD and 27 siblings, which were subsequently analyzed, 10 patients were taking a pharmacological treatment at the time of blood drawing. The M:F ratio was 5.7:1 (n = 23 male pairs, and n = 4 female pairs). A maximum ASD-SIB within-pair age difference of ± 2 years for pre-puberal pairs (< 8 years old) and ± 4 years for post-puberal pairs (≥ 8 years old) was allowed. The number of pre-puberal and post-puberal pairs was 13 (48.1%) and 14 (51.9%, respectively. ASD was diagnosed in affected siblings according to DSM-5 criteria^1^, and confirmed by ADOS or ADOS-2^61^ and ADI-R^62^. Unaffected siblings were screened clinically and found not to satisfy DSM-5 criteria for ASD. Autistic behaviors, adaptive functioning and intelligence quotient (IQ) were assessed in ASD patients, as previously described^63^. All parents gave written informed consent for their children. The consent form and experimental protocol were approved by the Institutional Review Board of University ‘‘Campus Bio-Medico’’ (prot. 14/98 and 30/14) and the ethics committee of the University of Messina (prot. 22/17). All methods were performed in accordance with the relevant guidelines and regulations.

### RNA extraction and RNA sequencing

Peripheral blood was drawn into Tempus blood RNA tubes (ThermoFisher) from all participants, and RNA was extracted using Tempus Spin RNA Isolation Kit following the manufacturer’s instructions. RNA quality was assessed by Bioanalyzer 2100 System (Agilent Technologies). All samples showed RIN values between 7.8 and 9.4. RNA-seq was conducted at the Center for Translational Genomics and Bioinformatics (San Raffaele Scientific Institute, Milan, Italy). Sequencing libraries were prepared with 250 ng of total RNA using TruSeq Stranded mRNA (Illumina, Inc.) following the manufacturer’s protocol. This approach delineates the coding transcriptome using Oligo-dT beads to capture polyA mRNA tails. The final library was sequenced by 100 bp paired-end sequencing on NovaSeq to generate 40 million clusters per sample.

### Data analysis

Quality controls on FASTQ files were conducted using FastQC software (http://www.bioinformatics.babraham.ac.uk/projects/fastqc/). Reads were aligned to the Human reference genome (GRCh37) using STAR v2.5.3a^64^. The featureCounts 1.6.2^65^ from the Subread package v1.4.6^66^ was next used to summarize and quantify the mapped reads, by counting the number of reads overlapping Ensemble gene annotations (homosapiens.grch37.75.gtf). We sequenced approximately 40 million reads per sample. Reads mapping was between 81.4 and 94.0% of total reads aligned, about 60% of these reads originating from CDS-exonic regions. Filtering was performed to remove genes with less than 10 total read counts across all samples. Data were variance stabilized using DESeq2 (*vst* function), and then Principal Component Analysis (PCA) was performed to assess the presence of outliers and to detect any batch effects. Gene expression differential analyses between proband and sibling were conducted by means of the R package DESeq2 v1.24.0^67^ using both unpaired and paired models. In the unpaired model, we correct for batch and pharmacological treatment. In the paired model, we did not correct for batch since each autistic individual was compared to his/her unaffected sibling, sequenced in the same batch. The *p* values were corrected for multiple testing using the False Discovery Rate (FDR) method with the Benjamini–Hochberg correction, considering as significant all the genes with adjusted *p* value (adj *p*) < 0.05.

### Weighted correlation network analysis (WGCNA)

WGCNA is based on the concept of a scale-free network with the presence of a few highly connected nodes (hubs) with many others poorly connected nodes. Detecting modules of co-expressed genes is one of the objectives of WGCNA, which was conducted using the WGCNA R-package^25,68^. Modules were visualized using Cytoscape, an open source software platform for visualizing molecular interaction networks and biological pathways, while integrating these networks with annotations, gene expression profiles and other state data (https://cytoscape.org/index.html)^31^. The gene expression matrix was composed of 18,982 genes after data preprocessing. Subsequently, counts data were filtered excluding genes with less than 10 counts across all samples. Then, they were variance stabilized using DESeq2 (*vst* function) and adjusted for batch and pharmacological treatment using the function “removeBatchEffect” as implemented in the limma R-package^69^. Low-variability genes (lower 50%) were excluded after being ranked by Minimum Absolute Deviation (MAD), obtaining a total of 9491 informative genes. We generated an unsigned co-expression network using the function blockwiseModules, with the option mergeCutHeight = 0.25. Then, we computed the module eigengenes and we investigated their relationship with disease status using a linear model, as implemented in the limma package, running a paired and unpaired model as for the differential expression analysis. Relevant coexpression networks were exported and visualized using Cytoscape v3.7.2^70^. Modules associated with disease status were further investigated using GO enrichment analysis using specific functions present in the Bioconductor clusterProfiler v 4.2.2 package^71^. The receiver operating characteristic (ROC) curve was computed for the functionally relevant modules using the WGCNA eigenvalues by means of the *roc* function, as implemented in the R-package *pROC*^72^.

## Supplementary Information

Supplementary Information 1.

Supplementary Information 2.

## Data Availability

The datasets generated and/or analysed during the current study are available in the Gene Expression Omnibus (GEO) repository (https://www.ncbi.nlm.nih.gov/geo/) [Accession Number to Datasets: GSE212645].
