# Supplementary material for: RNA sequencing of blood from sex- and age-matched discordant siblings supports immune and transcriptional dysregulation in autism spectrum disorder
Source: Sci Rep. 2023 Jan 16;13:807. doi: 10.1038/s41598-023-27378-w (PMC9842630; doi:10.1038/s41598-023-27378-w)
Supplement: Supplementary file 1 — Supplementary Information 1. [file 41598_2023_27378_MOESM1_ESM.docx]

Supplementary Information

**RNA sequencing of blood from sex- and age-matched discordant siblings supports immune and transcriptional dysregulation in Autism Spectrum Disorder**

Pasquale Tomaiuolo,^1^ Ignazio Stefano Piras,^2^ Simona Baghai Sain,^3^ Picinelli Chiara,^1^ Baccarin Marco,^1,4^ Castronovo Paola,^1^ Marco J. Morelli,^3^ Lazarevic Dejan,^3^ Maria Luisa Scattoni,^5^

Giovanni Tonon,^3^ and Antonio M. Persico,^6*^

^1^ Mafalda Luce Center for Pervasive Developmental Disorders, Milan, Italy

^2^ Neurogenomics Division, The Translational Genomics Research Institute, Phoenix, Arizona, USA

^3^ Center for Translational Genomics and Bioinformatics, IRCCS San Raffaele Scientific Institute, Milano, Italy

^4^ Synlab Suisse SA, Department of Genetics, Bioggio / Switzerland

^5^ Research Coordination and Support Service, Istituto Superiore di Sanità, Rome, Italy

^6^ Child & Adolescent Neuropsychiatry Program, Modena University Hospital & Department of Biomedical, Metabolic and Neural Sciences, University of Modena and Reggio Emilia, Modena, Italy

***** **Corresponding Author:** Antonio M. Persico, Prof. of Child & Adolescent Neuropsychiatry, Department of Biomedical, Metabolic and Neural Sciences, University of Modena and Reggio Emilia, via Giuseppe Campi 287, I-41125 Modena, Italy; email antonio.persico@unimore.it.


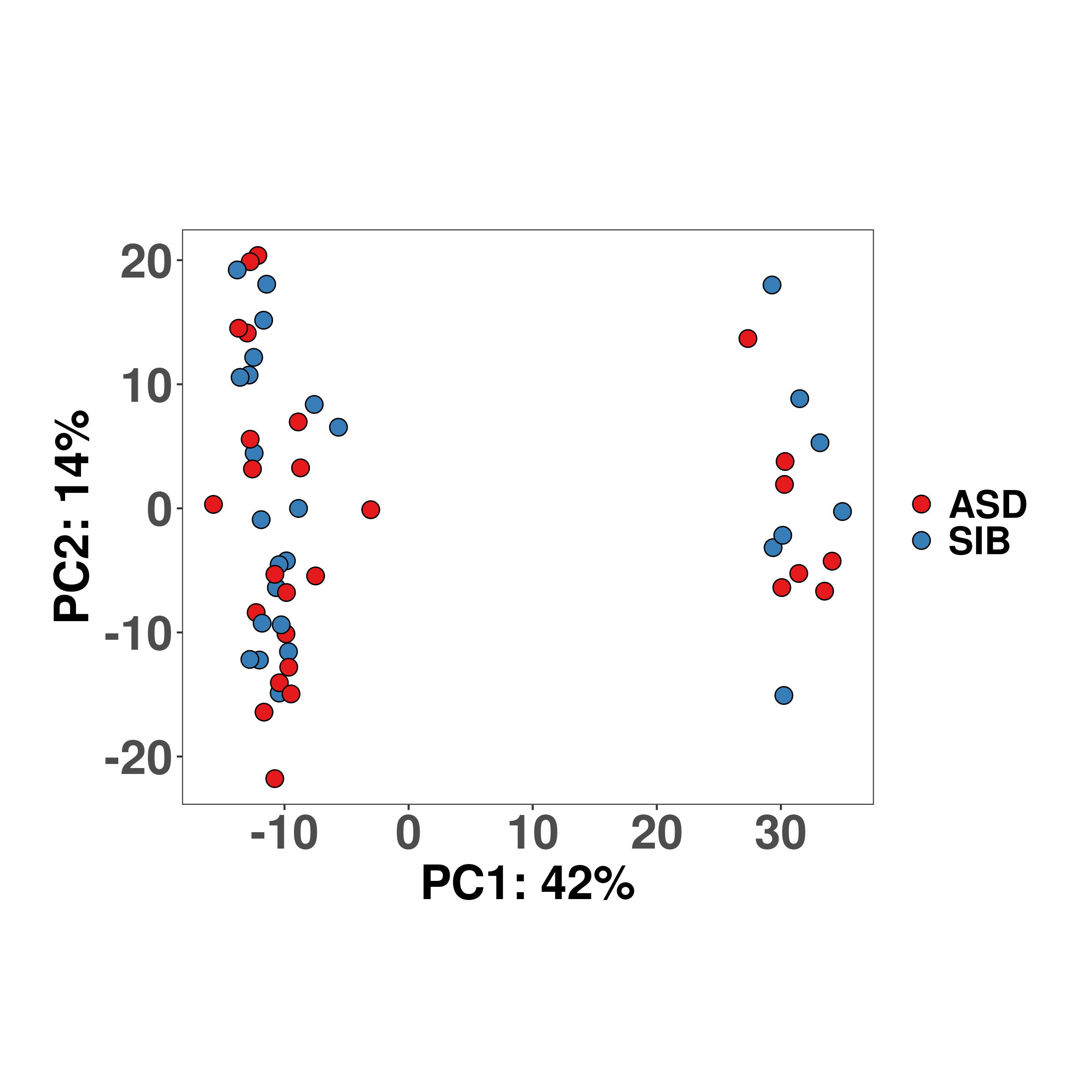


A

**
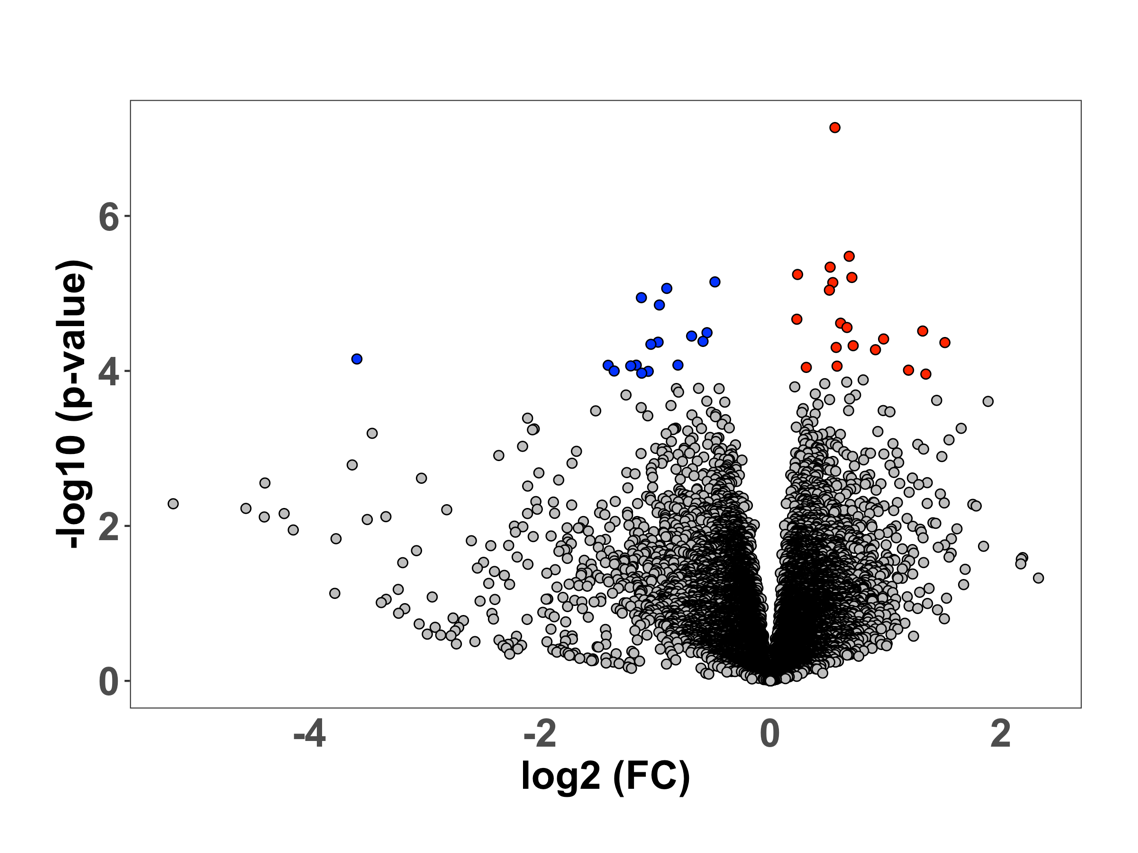
**

B

**Supplementary Figure S1A. PCA plot of RNA-seq data**.

Scatterplot of the top two principal components, highlighting the variability in PC1 between the two sequencing runs.

**Supplementary Figure S1B. Transcriptional effects of pharmacological treatment.**

Volcano plot of differentially expressed genes in 10 ASD patients receiving psychopharmacological treatment vs the remaining 17 ASD patients. Differentially expressed genes are highlighted in red (upregulated in children receiving drug treatment) or in blue (downregulated).

| gene | baseMean | log2FoldChange | lfcSE | stat | p-value | p-adj |
| --- | --- | --- | --- | --- | --- | --- |
| OR2W3 | 160.381 | 0.741 | 0.229 | 3.232 | 0.0012 | 1.000 |
| CD80 | 26.642 | 0.507 | 0.161 | 3.158 | 0.0016 | 1.000 |
| ZNF586 | 644.091 | 0.172 | 0.056 | 3.070 | 0.0021 | 1.000 |
| EGR2 | 35.880 | 0.584 | 0.191 | 3.056 | 0.0022 | 1.000 |
| RCVRN | 6.374 | 1.034 | 0.347 | 2.975 | 0.0029 | 1.000 |
| LIPC | 54.483 | -0.675 | 0.228 | -2.964 | 0.0030 | 1.000 |

**Supplementary Table S1.** Top genes differentially expressed between ASD and typically-developing siblings using the paired model. No gene retained statistical significance after FDR correction.


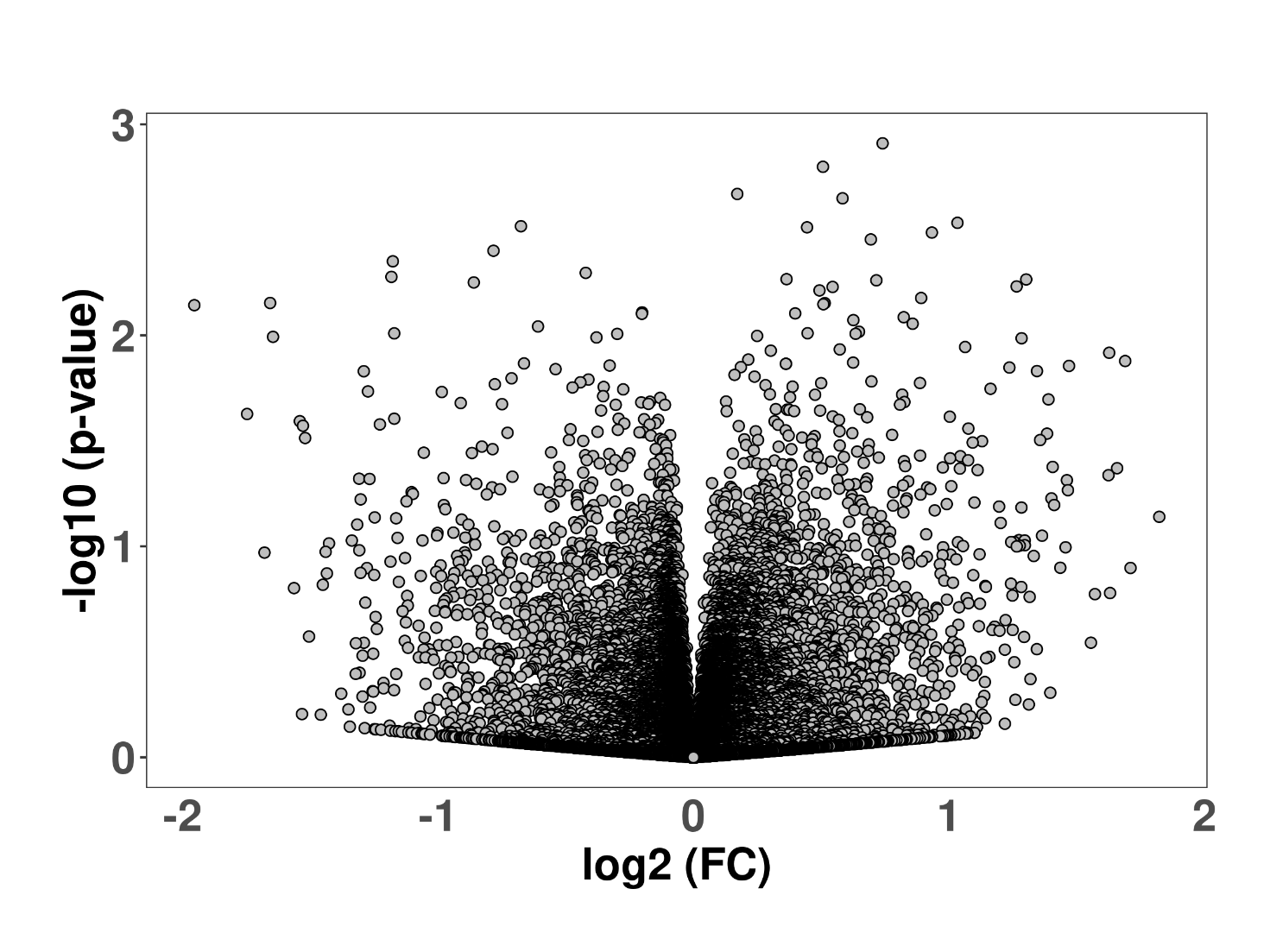


**Supplementary Figure S2*.*** Volcano plot of the transcriptional differences between ASD cases and typically-developing siblings using the paired model. No significant differentially expressed genes were detected.


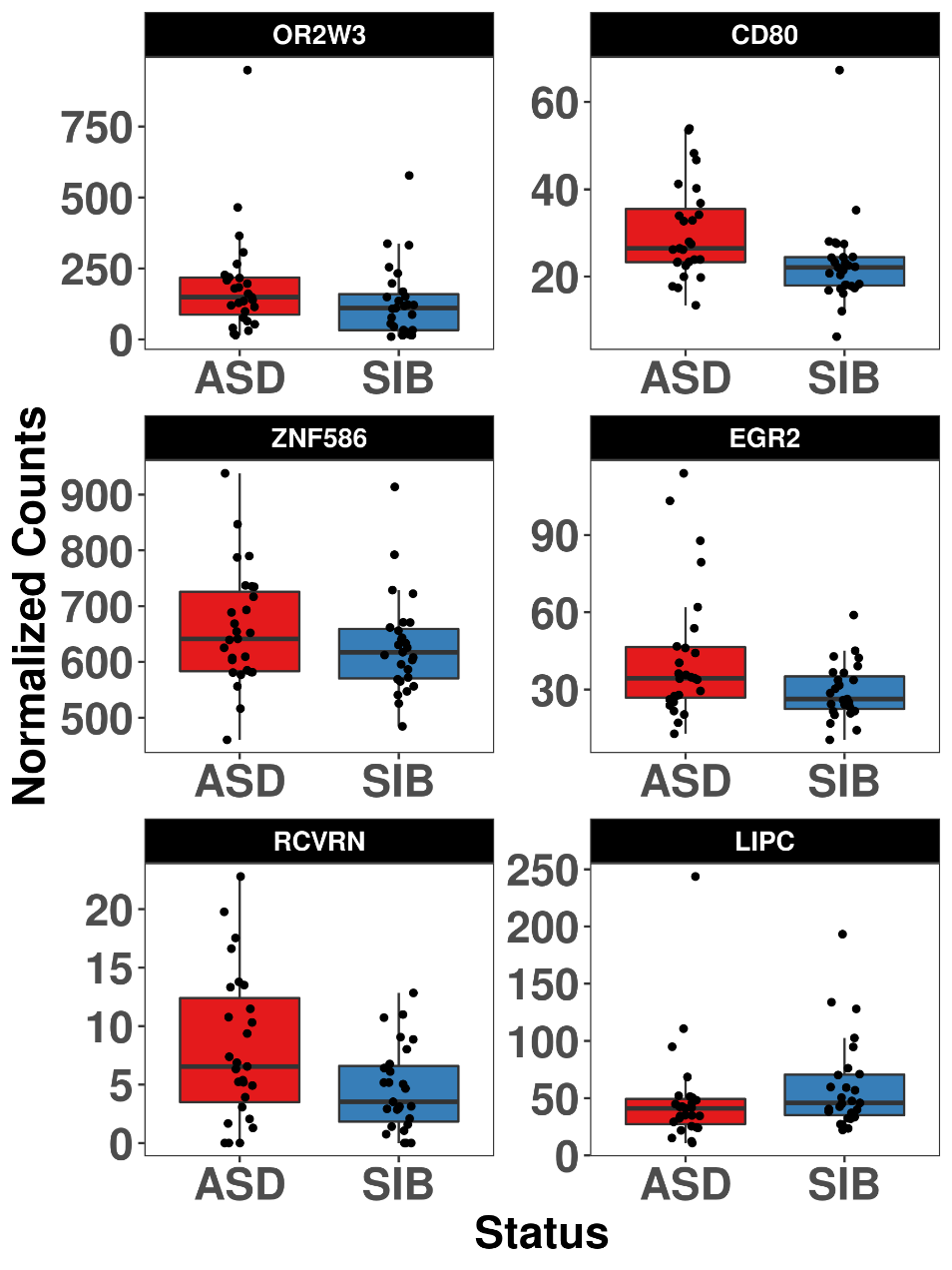


**Supplementary Figure S3.** Boxplot of the top six differentially-expressed genes obtained using the paired model and listed in Supplementary Table S1.

*
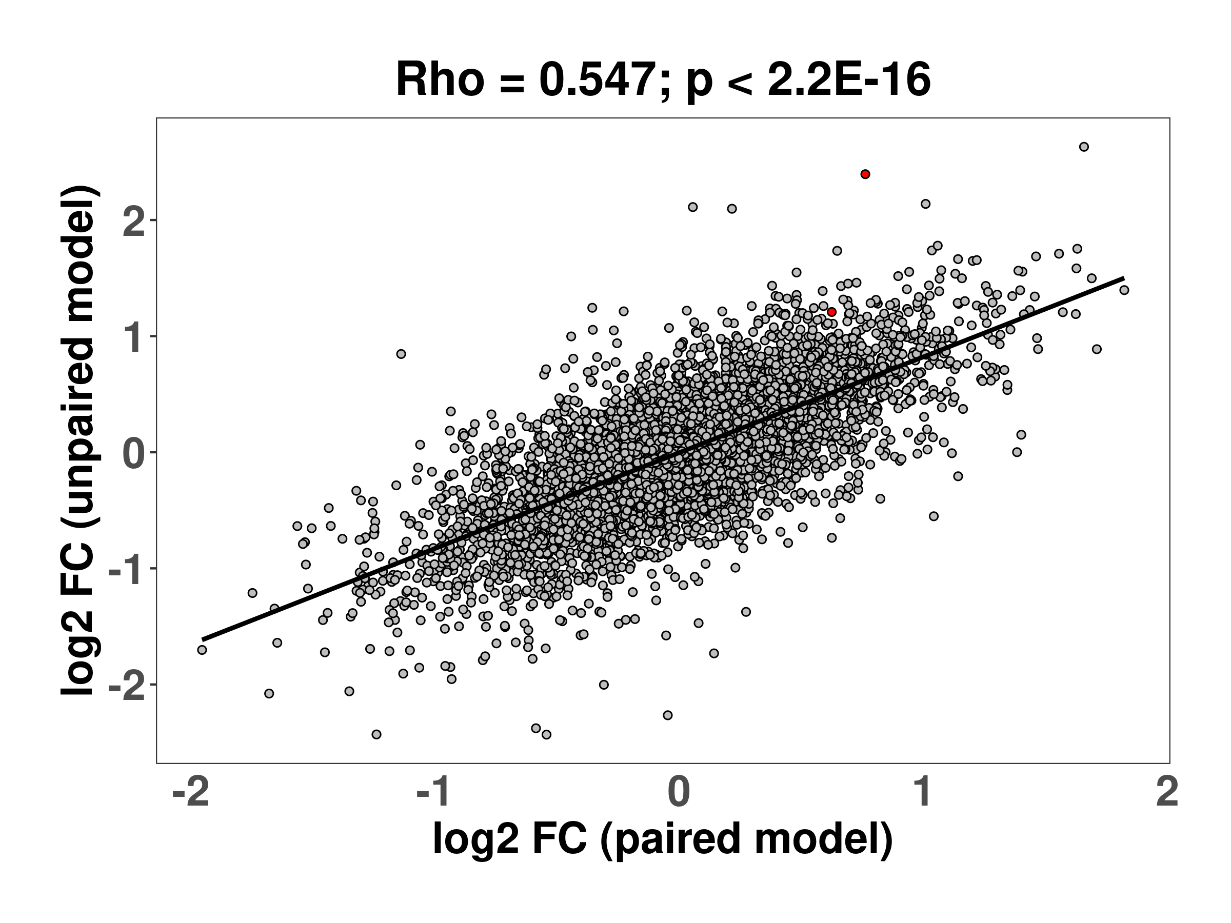
*

**Supplementary Figure S4.** Scatterplot showing the correlation between log2 FC (paired and unpaired model) from the genome-wide differential expression analysis.

**Supplementary Figure S5**. Differential expression patterns of all the module eigengenes in ASD vs SIB, ranked by p-value.

*
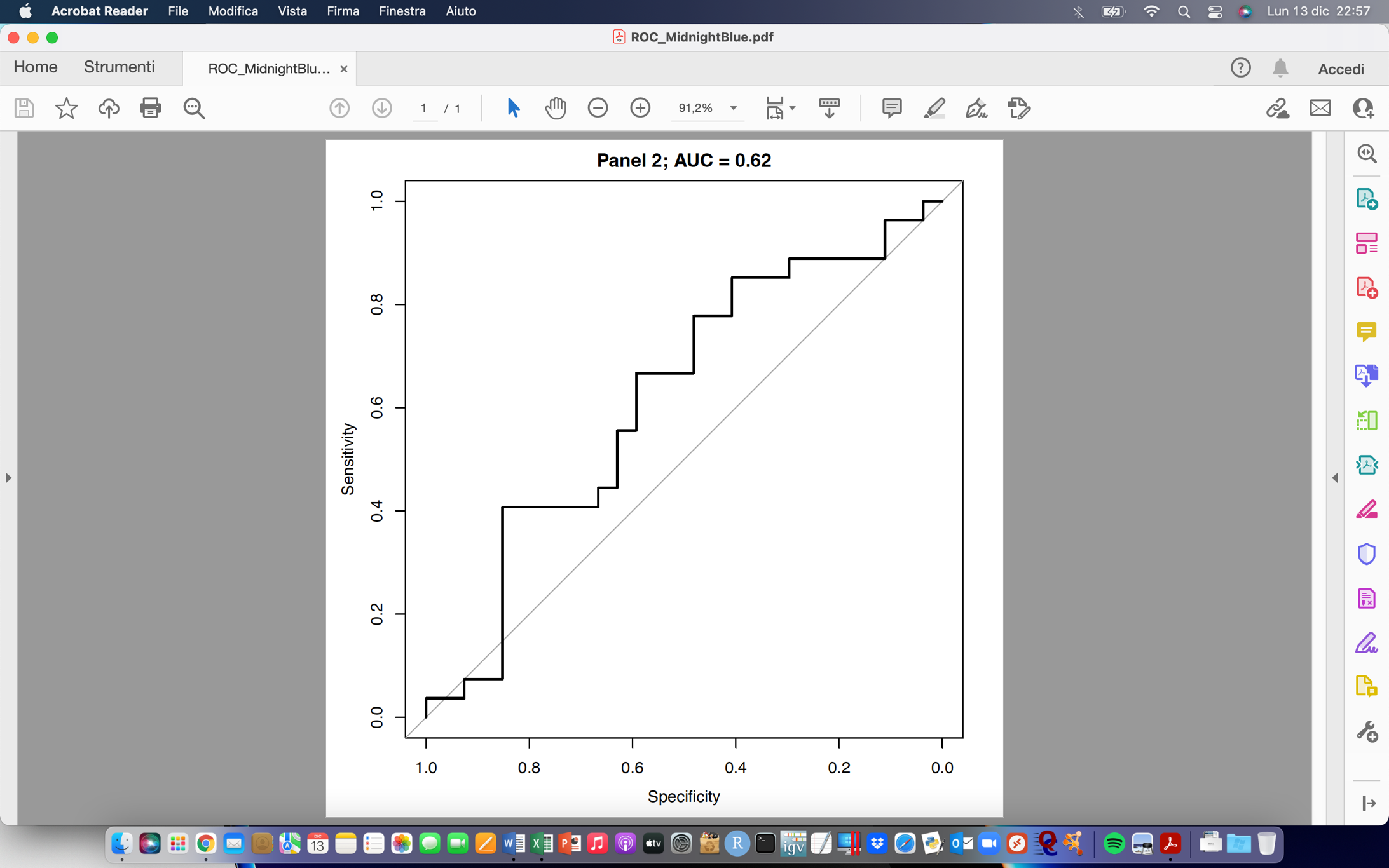
*

**Supplementary Figure S6.** ROC curve obtained using the “Midnight blue” module eigengene.

Supplementary Methods

WGCNA analysis – definition of terms

In a **scale-free network**, the connectivity among genes, pk, follows a power law distribution, pk ∼ k−gamma. One key feature of a scale-free network is the existence of a few highly connected nodes that participate in a very large number of metabolic reactions. With a large number of links, these hubs integrate all substrates into a single, integrated web. Scale-free networks have been shown to be robust against accidental failures, but vulnerable to coordinated attacks.

**Soft thresholding** is a value used to power the correlation of the genes to that threshold, under the assumption that raising the correlation to a power will reduce the noise of the correlations in the adjacency matrix. To pick up one threshold use the pickSoftThreshold function, which calculates for each power if the network resembles a scale-free graph. The power which produces a higher similarity with a scale-free network in our dataset was found to correspond to 8.

The **adjacency matrix** encodes the connection strength between each pair of nodes. The adjacency matrix indicates whether or not a pair of nodes is connected, i.e. its entries are 1 or 0. The adjacency matrix is the foundation of all subsequent steps. In particular, it is used to define node connectivity.

The purpose of **topological overlap matrix** is to better estimate the "connectedness" between two nodes/genes, rather than merely correlation from the similarity matrix.
